# Mental health and social relationships shape the work-from-home experience: lessons from COVID-19 pandemic

**DOI:** 10.3389/fpubh.2025.1526885

**Published:** 2025-04-10

**Authors:** Pierpaolo Mincarone, Carlo Giacomo Leo, Stanislao Fusco, Sergio Garbarino, Roberto Guarino, Antonella Rissotto, Maria Rosaria Tumolo, Giuseppe Ponzini, Egeria Scoditti, Saverio Sabina, Antonella Bodini

**Affiliations:** ^1^Institute for Research on Population and Social Policies, National Research Council, Brindisi, Italy; ^2^Institute of Clinical Physiology, National Research Council, Lecce, Italy; ^3^Training and Welfare Unit, National Research Council, Roma, Italy; ^4^Department of Neurosciences, Rehabilitation, Ophthalmology, Genetics and Maternal-Infant Sciences, University of Genova, Genova, Italy; ^5^Department of Biological and Environmental Sciences and Technology, University of Salento, Lecce, Italy; ^6^Institute for Applied Mathematics and Information Technologies “E. Magenes”, National Research Council, Milan, Italy

**Keywords:** forced work from home, healthy lifestyle, new normal, physical and mental health, work experience measure, family-to-work conflict, work-life balance

## Abstract

**Background:**

The great “work-from-home experiment” prompted by the pandemic has left an indelible mark both at the individual level—shaping expectations around life, work, and career—and at the organizational level. Evidence suggests that organizational success and performance are highly dependent on employee health and well-being, which contribute to higher productivity and engagement.

**Aim:**

This study aims to (1) examine the association between changes in depression severity and the work experience evaluation given by the staff of a large Italian research institute at the end of the forced telework period, and (2) explore the literature to link our findings to relevant recommendations for a more sustainable model of “new normal” work practices.

**Methods:**

A retrospective evaluation of validated health-related instruments was conducted following an 18-month period of enforced home working. In two subgroups defined by pre-pandemic depression severity (as assessed by the 9-item Patient Health Questionnaire), a multiple logistic regression analysis was conducted, adjusted for the influence of various individual, organizational, and psychophysical factors. Subgroup analysis was performed to explore potential differences in predictors of negatively perceived work experiences.

**Results:**

Pre-pandemic depression severity was not associated with perceived work experience (*p* = 0.60). In the subgroup of 244 participants with a pre-pandemic 9-item Patient Health Questionnaire total score of >4 (mild or greater severity), the main predictors of a negative work experience were a failure to reduce depression severity to the minimal level (aOR: 5.3, 95% CI: 2.23–14.29) and negative changes in interpersonal relationships within the family or among friends (aOR: 6.55, 95% CI: 3.05–14.78). In the subgroup of 489 participants with a pre-pandemic total score of ≤4 (minimal severity), the main predictors of a negative work experience were increased depression severity above the minimal level (aOR: 5.35, 95% CI: 2.74–10.64) and negative changes in interpersonal relationships within the family or among friends (aOR: 9.22, 95% CI: 5.16–17.00). The effect of worsened depression severity was modified by the availability of a dedicated workspace at home.

**Conclusions:**

These findings underscore the need for workplace interventions targeting not only clinical but also subclinical depression, with special attention to remote workers. Such efforts can benefit both individual well-being and the broader work environment. The importance of interpersonal dynamics within family and social networks was also confirmed, reinforcing the need for a work-life balance culture embraced by both employers and employees.

## Graphical Abstract

Graphical representation of risk factors (red) and protective factors (green) for a negative work experience for each subgroup. 
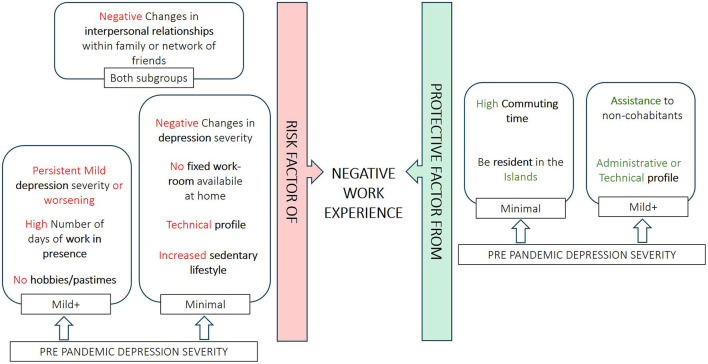

## 1 Introduction

The widespread adoption of remote work, one of the most significant organizational changes resulting from the COVID-19 pandemic (1), has revealed public attitudes toward this work arrangement (2). As anticipated by media outlets (3) and reflected in surveys (2), the gradual decline of the pandemic has not led to the elimination of remote work. On the contrary, organizations and workers are currently exploring diverse methods to establish a “new normal” that incorporates remote work practices (4, 5).

The potential impacts of remote work extend beyond the workplace into personal life domains, as the increased organizational flexibility and reduced commuting time associated with remote work can enable more family time, more personal time, healthier lifestyles. This setup creates more opportunities for employees to adopt healthy habits and engage in recreational activities, ultimately helping to reduce work-related stress (6). Furthermore, remote work enables employees to better synchronize working hours with family responsibilities, potentially decreasing time-based conflicts (7). The pandemic has indirectly made these opportunities widespread, setting the stage for leading employees to reassess their lives and their work based on how well it aligns with their desired lifestyles (8). Therefore, the so-called “great work-from-home experiment” (9) brought about by the COVID-19 pandemic provided a unique chance to examine how lifestyle, health, and social relationship changes can shape the work experience.

The effects of working from home on individual well-being, work-family conflict and job satisfaction have been predominantly studied in subjects who were able to voluntarily select this working modality. Instead, the pandemic has forced teleworking and this has even prompted a review of traditional models of studying work-family conflict (10), or to interpret the results of the models differently in light of the peculiarity of the situation (11).

As social distancing was imposed in addition to the requirement to work from home, workers experienced a reduction in both of their main resources for managing work-related stress: work-related social support (colleagues, supervisors) and non-work-related social support (family and friends). Given that social interactions play a fundamental role in the acquisition of personal resources, according to the conservation of resources theory the depletion of social relationship can yield emotional exhaustion, stress, and reduced job engagement (12, 13). Non-work social support in particular could be more strongly related to general health and well-being than to specific work-related strains (12, 14).

A large body of literature demonstrates that health and well-being are critical factors for individual job performance, organizational success (15), and work engagement (16). The close interconnection between mental health and work is particularly well-documented, even among academic professionals (17). Recent estimates indicate that on average 12% of adults have experienced depression at some point in their lives (18), a condition that profoundly impacts work productivity through both absenteeism and reduced work participation and functioning (19–21). Increases in mental health issues have been reported among workers who typically worked in offices prior to the pandemic but were required to work from home due to government or company policies (22, 23), with studies suggesting the persistence of these effects over time (24).

In this data-driven study we aimed to examine the association between changes in self-reported depression severity and ratings of work experience collected via an online survey among the staff of the Italian National Research Council (CNR), the largest public research institution in Italy. The survey was conducted at the end of the 18-month period of forced remote working and just before the regulated ordinary introduction of voluntary smart working, to collect information on health and lifestyle changes that may have occurred in a work context that had essentially never experienced remote working before. The main changes that emerged in health and lifestyle have been described in our previous studies (25–27). Here we build on those findings with a focus on changes in depression severity. This study aims to understand whether and to what extent these changes have influenced the perception of the work experience in those 18 months. In particular, planned subgroups analyses based on pre-pandemic depression severity were considered to provide a more differentiated assessment of the predictors of the perceived experience. Furthermore, we explored the literature to link our findings to relevant recommendations for a more sustainable model of “new normal” remote work practices.

## 2 Material and methods

### 2.1 The study

The survey was based on a retrospective assessment using validated health-related instruments. 748 individual validated questionnaires were collected online from CNR fixed-term or permanent workers who had been hired at least 6 months before the pandemic. In addition to considering socio-demographic variables and factors at the individual and familiar level potentially impacting the remote working experience, the survey extensively explored health-related aspects relevant to work performance. Diet, sleep quality and depression symptoms were investigated in detail using the Italian version of self-reported screening tools validated for the general population: The Mediterranean Diet Adherence Score (MEDAS) (28), the Pittsburgh Sleep Quality Index (PSQI) (29, 30), the Epworth Sleepiness Scale (ESS) (31–33), and the full Patient Health Questionnaire (the 9-item depression module, PHQ-9, and the single item asking for the degree of difficulty that any of the problems eventually checked out in the PHQ-9 brought to the work, personal and social spheres) (34–37). Employees were asked to fill in these questionnaires referring to both before and during the WFH period.

The impact of remote working on the work experience was investigated using an ad hoc developed and already validated one-dimensional measure, the Work Experience Measure (WEM) (25). The WEM score represents the mean of 7 items on a 5-point Likert-type scale, from 1 (very negative impact) to 5 (very positive impact). The items explore the perceived impact of WFH on the ability to take initiatives and propose solutions in the workplace, the participation in the working context, the relationship with colleagues and superiors, the quality of work, and the organization and management of personal environment, workspace and working time.

### 2.2 Ethical issues

The ethical approval was provided by the CNR Research Ethics and Integrity Committee (Ethical Clearance 0078918/2021). To ensure anonymity for colleagues, the implementation of the questionnaire and data collection were outsourced to an external company (eResult S.r.l., acting as a processor in accordance with the European General Data Protection Regulation 2016/679). Access to the survey was restricted exclusively to CNR staff through a unique token system. This token was provided solely via video (and not as plain text) to individuals who, through the institutional mailing system, expressed their interest in participating. eResult S.r.l. stored the email addresses and tokens separately from the questionnaire responses, retaining them only for the duration of the survey to prevent duplicate submissions. The raw response database—comprising solely pseudonymous survey responses—was then sent to the two authors authorized to process personal data for statistical analyses (AB, ES). Data transfer was protected using asymmetric encryption with dual public/private keys via an SSH File Transfer Protocol, secured by a username and password. Further details are provided in (25).

### 2.3 Variables of interest

For the purposes of this analysis, we focused on the binary outcome of negative (1 ≤ WEM < 3) vs. positive (3 ≤ WEM ≤ 5) impact of WFH on the work experience.

To better describe the changes that occurred during the period of WFH in MeDAS, PSQI, and PHQ-9 total scores, we introduced meaningful categorical changes for each indicator. Since excessive daytime sleepiness was reported by only the 5.4% of participants before the pandemic (a proportion significantly reduced to 3.1% during the WFH period, McNemar test *p-*value of 0.007), the ESS was not considered in this analysis.

For sleep or depressive disorders, relevant changes were defined by referring to the normative thresholds of the two scales (26, 27). The choice of the lower normative thresholds for both the PSQI and PHQ-9 total scores was aimed at bringing together without distinction all the participants who could present indications of possible disorders, because discussion of cases with possible clinical implications was beyond the scope of the study. For interpretative convenience, we will use the terms “improvement” and “worsening” exclusively to indicate the passage through the thresholds. Instead, in the case of MEDAS, we considered the failure to increase the score as a single negative change in eating habits (see Supplementary Table 2).

Regarding the single-item question in the PHQ, 92.8% of the 613 participants who checked off any problems both before and during the WFH period reported that these problems made somewhat difficult (and no more) to do their work, take care of things at home or get along with other people. Therefore, in this analysis the categorical variable indicating the four possible combinations of absence (Not difficult at all) and presence (any of the other possible answers) of difficulty (before vs. during) was considered for those 613 participants.

Among the psycho-physical indicators we also considered weight change, changes in sedentary lifestyle and in the engagement in hobby/pastime, and the perceived impact of WFH on the interpersonal relationships within the family and the network of friends (see Supplementary Table 3).

### 2.4 Statistical analysis

The analysis was conducted on 733 (of 748) participants with complete data. The two subgroups of participants defined by pre-pandemic depression severity were the subgroup “Minimal” consisting of 489 participants with minimal depression severity (PHQ-9 ≤ 4), and the subgroup “Mild+” including 244 participants with mild or more severe depression (PHQ-9 > 4). The chi-square test with normal-based analysis of residuals was used to assess the association of the above defined categorical variables with the impact of WFH on the work experience and to identify those specific cells contributing the most to the results. Having established the association between the variation of PHQ-9 and the evaluation of the impact of WFH on the work experience in both subgroups, a simple moderation analysis was carried in each subgroup to test whether the direction and strength of the relationship are influenced by the socio demographic variables, work-related factors (profile, commuting time, number of days in presence), family factors (size of the house, number of family members sharing the same accommodation, the number and age of children in the household and the presence inside and outside the home of people in need of assistance), individual organizational factors related to the working space available in the home, and by health-related factors.

Finally, multiple logistic regression analysis was used to examine the statistical significance of the association between health-related variables and the impact on the work experience after adjusting for the influence of the other individual, familiar and organizational factors. Any variable with at least weak significant association (*p* ≤ 0.20) was included in the initial multiple logistic regression model. Given the overlap of the single-item question of the PHQ with the dependent variable, the related data were not considered in the regression analyses. A backward model selection based on AIC was carried out. Multicollinearity was assessed by computing the variance inflation factor.

All analyses were performed using the statistical software R and the packages available therein (38). Model diagnostics were computed using the R packages car (39), pROC (40), and ResourceSelection (41).

## 3 Results

### 3.1 General characteristics of subgroups

The general demographic and working characteristics of the study population have been presented in (25–27) and summarized in Supplementary Table 1 for ease of consultation.

Nearly a third of the participants (29.1%) reported an overall negative impact (1 ≤ WEM < 3) of WFH on the working experience.

In Table 1 the Minimal and Mild+ subgroups are compared with respect to the main socio-demographic and professional characteristics. The percentage of participants for whom the impact of WFH on their work experience was negative (WEM < 3) was not significantly different in the two subgroups (29.7% vs. 27.5%, *p* = 0.60). Among the individuals in the Mild+ subgroup there are significantly fewer participants over 60 years of age, more women, more technicians and fewer researchers than in the Minimal subgroup.

**Table 1:** Comparison of the two subgroups with respect to the main socio-demographic and professional characteristics.

**Factors**		**Response options**	**Minimal subgroup 489 participants**	**Mild+ subgroup 244 participants**	*p-*value^*^
Work environment	Perceived impact of WFH on the work experience	Positive (WEM ≥ 3)	344 (70.3%)	177 (72.5%)	0.60
		Negative (WEM < 3)	145 (29.7%)	67 (27.5%)	
	Professional profile	Technician	96 (19.6%)	63 (25.8%)	0.05
		Administrative	44 (9.0%)	31 (12.7%)	
		Technologist	51 (10.4%)	25 (10.3%)	
		Researcher	298 (61.0%)	125 (51.2%)	
Demographic	Age	≤ 39 years	58 (11.9%)	29 (11.9%)	0.02
		40–49	190 (38.9%)	81 (33.2%)	
		50–59	168 (34.3%)	110 (45.1%)	
		≥60	73 (14.9%)	24 (9.8%)	
	Gender	Male	228 (46.6%)	83 (34.0%)	0.001
		Female	261 (53.4%)	161 (66.0%)	
	Macro-region of residence	North	155 (31.7%)	84 (34.4%)	0.40
		Center	179 (36.6%)	76 (31.2%)	
		South	110 (22.5%)	55 (22.5%)	
		Islands	45 (9.2%)	29 (11.9%)	
	Living alone	Yes	67 (13.7%)	37 (15.2%)	0.67
		No	422 (86.3%)	207 (84.8%)	

^*^Chi-square test.

### 3.2 Bivariate analysis in subgroups

The entire bivariate analysis in subgroups is reported in Supplementary Table 4.

#### 3.2.1 Minimal subgroup

Of the 489 participants in the Minimal subgroup (pre-pandemic PHQ-9 ≤ 4), 145 (29.7%) reported a negative assessment of the impact of WFH on work experience. All health-related variables show significant associations with the outcome. The analysis of residuals highlights that a negative impact on work experience is significantly more frequent among participants showing worsened depression (56.7% compared to 21.7% of participants who remained at the pre-pandemic level), worsened sleep quality (48.5% vs. ≤ 28.6%), a non-increased adherence to the Mediterranean diet (33.7% compared to 24.0% of those who increased it by even one point), an increase in weight (37.0% vs. ≤ 26.0%), in sedentary lifestyle (41.4% vs. ≤ 20.7%), a negative impact of WFH on interpersonal relationships within family or network of friends (73.4% vs. 19.2%), and among those who lost hobbies and pastimes (50.1% vs. ≤ 29.1%). Finally, among those participants who reported that the presence of depressive symptoms brought some difficulties in their work, personal or social spheres during the WFH, the percentage of a negative impact on the work experience is significantly higher. This occurs regardless of pre-pandemic conditions (≥50.0% vs. ≤ 20.7%). It should be underlined that 96.6% of the participants who reported the disappearance of these difficulties during the WFH period also reported a positive impact on their work experience.

The moderation analysis showed that the association between worsened depression and negative work experience was significantly stronger when subjects lived in a small house (see Supplementary Figure 1). No other moderating effects were found.

#### 3.2.2 Mild+ subgroup

Of the 244 participants with complete data in the Mild+ subgroup, 67 (27.5%) resulted in a negative assessment of the impact of WFH on work experience. This proportion is not significantly different from that observed in the Minimal subgroup. The analysis of residuals highlights that the fraction of participants who reported a negative impact on work experience is significantly lower among those improving their depressive condition, compared to participants who remained in the same condition (10.4% vs. 35.3%). Even in this subgroup, the frequency of negative impact on the work experience is significantly higher among the individuals reporting difficulties due to depressive symptoms in daily activities during the WFH period (≥35.7% vs. ≤ 20.9%).

Unlike the Minimal subgroup, there is no significant association between the assessed impact of WFH on work experience and the changes in adherence to the Mediterranean diet (*p* = 0.48). The association with changes in sleep quality is only weak (*p* = 0.053), with a similar trend to that reported for the Minimal subgroup. The percentage of the negative outcome is indeed highest among participants who have worsened their condition from good to poor sleep quality (52.9% vs. ≤ 29.7%), but these participants are only 17. In the Mild+ subgroup, the outcome is only weakly associated with the change in weight (*p* = 0.11), while the significant association with the change in sedentary lifestyle already highlighted in the Minimal subgroup is maintained (*p* = 0.002). Even in the Mild+ subgroup, the negative outcome is significantly more frequent among participants with an increased sedentary lifestyle (37.3% vs. ≤ 17.5%) and among those reporting a negative change in the interpersonal relationships within family or friends (59.2% vs. 19.5%). Finally, the analysis of residuals suggests that individuals who engaged in hobbies and pastimes during the WFH period (in continuity with the past or for the first time) less frequently than the others reported a negative impact on the work experience ( ≤ 22.8% vs. ≥43.6%).

No moderation effect was found in this subgroup.

### 3.3 Multiple logistic regression analysis in subgroups

The graphical summary of the results of the multiple logistic regression analysis in the two subgroups is presented in Graphical abstract.

#### 3.3.1 Minimal subgroup

A preliminary multiple logistic regression model included variables from individual, familiar, and individual organizational factors such as commuting time, macro-region, size of the municipality of residence, professional profile, number of days of work in presence, availability of a fixed work-room at home, frequency of sharing the work-room (*p* < 0.05), and size of the house (*p* = 0.12). The included health-related variables were weight change, changes in sedentary lifestyle, changes in engagement in hobbies and pastimes and the variables expressing changes in MEDAS, PSQI and PHQ-9 as indicated in Supplementary Table 4. A sensitivity analysis on the levels of the considered factors led to the definition of a slightly more parsimonious model with seven predictors, as described in Table 2. Five of them (availability of a fixed work-room at home, commuting time, changes in sedentary lifestyle, professional profile, and macro-region of residence) have been already evidenced in Bodini et al. (25) as relevant individual, familiar, and individual-organizational factors influencing lifestyle and work experience during the WFH period. Two health-related factors are now highlighted: changes in depression severity and changes in the interpersonal relationships within family or network of friends. The interactions of these two variables with the other in the main model were also examined. Only the interaction between availability of a fixed work-room at home (“Yes”/“No”) and changes in depression severity was significant (*p* < 0.05) and therefore included in the model, as reported in Table 3. The goodness of fit assessed with the Hosmer–Lemeshow test is good (*p* = 0.73) and an excellent discrimination capability is estimated by the area under the ROC curve (AUC = 0.8451, 95% CI: 0.8063–0.884 with the DeLong method). The model was not affected by multicollinearity. Although few covariates were significantly correlated, the variance inflation factor did not raise significant concerns of collinearity.

**Table 2:** Odds ratios (ORs) and 95% confidence intervals (CIs) of an overall negative impact of WFH on the work experience (1 ≤ WEM < 3) calculated by multiple logistic regression analysis in the Minimal subgroup.

**Variable**	**OR**	**95% CI**
**Changes in interpersonal relationships within family or network**		
**of friends**		
Null or positive	1.00	
Negative	**9.22**	5.16–17.00
**Changes in depression severity**		
Unchanged, PHQ-9 ≤ 4	1.00	
PHQ-9 from ≤ 4 to > 4	**5.35**	2.74–10.64
**Availability of a fixed work-room at home**		
Yes	1.00	
No	**2.32**	1.19–4.49
**Commuting time**		
≤ 15 min	1.00	
15–30 min	0.62	0.33–1.16
>30 min	**0.22**	0.12–0.42
**Changes in sedentary lifestyle**		
Unchanged	1.00	
Decreased	1.41	0.57–3.29
Increased	**2.11**	1.25–3.62
**Professional profile**		
Researcher	1.00	
Technologist	0.75	0.30–1.74
Administrative staff	0.47	0.17–1.20
Technical staff	**2.35**	1.28–4.35
**Macro-region**		
North	1.00	
Center	0.98	0.54–1.76
South	1.18	0.61–2.27
Islands	**0.31**	0.10–0.86
**Interaction**		
Absent		
Present	**0.27**	0.08–0.87

Significant ORs are shown in bold.

**Table 3:** Modification of the effect of availability of a fixed workroom at home and changes in depression severity in the minimal subgroup calculated by multivariable logistic regression analysis in the Minimal subgroup.

		**Change in depression severity**		**Effect of changes in depression severity within the strata of availability of a fixed work-room at home**
		**Unchanged, PHQ-9** ≤ **4**	**PHQ-9 from** ≤ **4 to** >**4**	
Availability of a fixed work-room at home	Yes	1	5.35 [2.72, 10.51]	5.35 [2.72, 10.51]
	No	2.32 [1.2, 4.49]	3.3 [1.37, 7.92]	1.42 [0.53, 3.83]

Data reported as OR [95% CI] of an overall negative impact of WFH on the work experience (1 ≤ WEM < 3).

WFH, working from home; WEM, work experience measure.

#### 3.3.2 Mild+ subgroup

The initial multiple logistic model included the following variables: changes in depression severity (reduction of PHQ-9 to ≤ 4 vs. remaining at the level >4), living alone, need of assistance to non-cohabitants, commuting time, professional profile, availability of a fixed work-room at home, engagement in hobbies and pastimes during the period of WFH (as from the bivariate analysis), changes in sleep quality, changes in the interpersonal relationships within family or network of friends, in weight, in sedentary lifestyle (*p* < 0.05), graduation, the size of the municipality of residence and number of days of work in presence (*p* ≤ 0.20). The main effects of the final estimated model are reported in Table 4. Even in this group, the variables related to variations in the severity of depression and in interpersonal relationships within family or network of friends are particularly significant. No significant interactions were found. The model passed the Hosmer-Lemeshow test for goodness-of-fit (*p* = 0.62), demonstrated an excellent discrimination capability (AUC = 0.8523, 95%CI: 0.8027–0.9019 by the DeLong method), and was not affected by multicollinearity.

**Table 4:** Odds ratios (ORs) and 95% confidence intervals (CIs) of an overall negative impact of WFH on the work experience (1 ≤ WEM < 3) calculated by multiple logistic regression analysis in the Mild+ subgroup.

**Variable**	**OR**	**95% CI**
**Changes in interpersonal relationships within family and friends**		
Null or positive	1.00	
Negative	**6.58**	3.05–14.78
**Changes in depression severity**		
PHQ-9 from >4 to ≤ 4	1.00	
Unchanged, PHQ-9 > 4	**5.30**	2.23–14.29
**Habit of hobbies/pastimes during WFH**		
Yes	1.00	
No	**3.46**	1.64–7.48
**Professional profile**		
Researcher or technologist	1.00	
Administrative or technical staff	**0.14**	0.03–0.54
**Availability of a fixed work-room at home**		
Yes	1.00	
No	1.85	0.84–4.01
**Assistance to non-cohabitants**		
No	1.00	
Yes	**0.44**	0.20–0.92
**Living alone**		
No	1.00	
Yes	2.08	0.84–5.12
**Number of days of work in presence**		
≤ 60	1.00	
>60	**2.25**	1.11–4.65

Significant ORs are shown in bold. No significant interactions were found.

WFH, working from home; WEM, work experience measure.

## 4 Discussion

The main finding of our study is the statistical association between even small changes in depression severity during the WFH period and the perceived impact of enforced WFH on work experience. This association is significant regardless of pre-pandemic depression severity. Changes in interpersonal relationships within family or friends also emerged as significant factors in both subgroup analyses.

In their critical review of the literature, de Oliveira and coauthors (42) recognize that studies on the relationship between mental health and worker productivity only consider the most common mental disorders and concluded that more high-quality, longitudinal and causal inference studies are needed to provide clear recommendations. Researchers and institutions are trying to develop substantial recommendations and identify best practices. Several systematic reviews and meta-analyses have explored the effectiveness of workplace interventions for diagnosed depression or anxiety (43, 44). The Luv U Project developed several recommendations for specific actions to be taken to advance mental health in the workplace based on a review of best and promising practices in US and the work of an Advisory Council (45, 46). Recommendations include to measure employee mental health and wellbeing outcomes. To develop an adequate quantitative process for measuring mental health in the workplace is indeed the first step toward the implementation and evaluation of corporate mental health promotion programs beyond the support to primary care interventions. This is also supported by WHO (19). Our study suggests that successful workplace interventions should also target subclinical conditions and that special attention should be given to remote workers. Indeed, both before and during the pandemic, over 90% of participants in our sample who exhibited symptoms fell into the subclinical range. Although most respondents did not show clinically important changes in PHQ-9 scores (i.e, changes of at least 5 points, (97), several subjects (34.5%) reported increased depression severity during the WFH period, typically on the order of 1-4 points, and these even small changes appear to have influenced the work experience. Health interventions for depression could be effectively implemented digitally for individuals with subclinical symptoms (47, 48), providing benefits not only to the individual but also to the work environment. The nine symptoms assessed by the PHQ-9 have been linked to both absenteeism and presenteeism (49–51)—the loss of productivity when workers are physically present but unable to perform at full capacity due to physical, mental, or emotional strain (20, 21, 43). The pandemic has heightened presenteeism, introducing the more invisible phenomenon of virtual presenteeism on a larger scale (52, 53). Organizations should therefore establish best practices to balance minimizing presenteeism—even in remote work settings (54)—with the positive effects that work itself can have on certain health conditions when conducted in supportive environments (55). Beyond primary care interventions, addressing workplace mental health requires shaping workplace cultures to value mental health and well-being, reducing stigma around mental illness, enhancing the dissemination and accessibility of workplace mental health information, providing training for managers, and supporting flexible solutions that enable employees to perform effectively (56). These measures can encourage workers to seek empathy and support from colleagues and professionals (57–59). Consistent with conservation of resources and self-determination theories, a supportive work environment allows an ill employee to maintain a level of productivity and also to proceed with the recovery of physical or mental health (55). This presents a new challenge: fostering a sense of belonging, social support, and a collaborative work culture among workers who do not physically share the same work and social spaces. Organizational climate (beliefs and values) profoundly influences attendance behavior, and an open question remains as to whether and how identification with in-person work norms may evolve in virtual environments (60).

In the subgroup of participants with mild or greater depression severity pre-pandemic, engagement in hobbies or pastimes was a protective factor against a negative work experience. Most of the recovery from work-related stress and fatigue usually occurs off-hours, between work shifts. Leisure time provides a wonderful opportunity to engage in creative activities, social contact and physical activity that contribute even more than passive rest to reducing work-related stress, allowing the restoration of depleted physical and psychological resources (61). Based on the Job Demands-Resources model, leisure crafting was found to be negatively related to emotional exhaustion in remote workers (62, 63). Therefore, subjective proactive action plans including hobbies can provide an alternative means for remote workers to establish a psychological boundary between work and personal life, compensating for the lack of physical separation and to improve the work experience. Furthermore, hobbies also play an essential role in maintaining a healthy balance (64), providing structured activities that facilitate the identification of physical or virtual spaces for relaxation and rejuvenation (65). Organizations can leverage employees' engagement in leisure crafting to help establish or restore boundaries during remote work. Emphasizing the value of meaningful leisure may be beneficial at the organizational level. For example, companies can create frameworks that encourage leisure crafting, such as providing financial support for diverse, stimulating leisure activities (62).

Living alone also emerged in our study as a risk factor for participants with mild or greater depression severity pre-pandemic. This may reflect demographic characteristics: participants living alone were predominantly women over 40 residing in large cities, for whom social isolation may have had a pronounced impact. For younger women, beginning their careers with remote work may support family planning (66). Recent studies indicate that Generation *Z*-values work flexibility and WFH options for work-life balance, regardless of gender (67). Given that remote work can exacerbate feelings of social isolation and loneliness, it is crucial to implement strategies that enhance team connectivity even in virtual settings (68). Informal virtual meetings have been shown to promote inclusivity, psychological safety, and trust among team members (69). Yet, excessive virtual meetings may contribute to burnout or disengagement, underscoring the need for a balanced approach (68). To mitigate these risks, it is important to clearly articulate the purpose of each meeting to avoid perceptions of increased workload (70). Moreover, mandatory 'Camera On' policies should be carefully evaluated, as they can lead to mental fatigue and contribute to burnout over time (71).

As mentioned, a negative impact of WFH on family and friend relationships was strongly associated with a negative work experience in both subgroups. This was expected, as the shift to remote work can disrupt the delicate balance between professional responsibilities and family obligations, presenting challenges that shape the work experience (72). The vast pre-pandemic literature on work-family conflict largely focused on work-to-family conflict. However, pre-pandemic studies have already highlighted the possibility of family-to-work but not work-to-family conflict among remote workers (73, 74). Since family-to-work conflict is an important predictor of job satisfaction, stress and burnout, and performance (75–78), this component of work-family conflict, albeit less studied in literature, has become of great interest from a work organization perspective. Using the Vulnerability-Stress-Adaptation model, Wu et al. (79) demonstrate the trade-off between family relationship quality and work-life balance, arguing that spending more resources such as energy and attention for family relationships reduced the perceived work-life balance. Accordingly, they recommend that organizations implement family-friendly policies. This means that employers should not just consider WFH in and of itself a family-friendly policy, but rather create a culture that supports the importance of personal time. Family-friendly policies for remote workers can include encouraging the use of calendar blocking to effectively manage work and personal commitments, for example. This approach helps establish clear boundaries while also improving work coordination in a transparent and respectful way. Another example of family-friendly policy emerges from a study integrating conservation of resources theory and effort-recovery model (80). The study found that breaks during remote work can be efficiently used to buffer the resource-depleting impact of interruptions for remote workers. However, taking breaks may be viewed as an inappropriate behavior and employees can be reluctant to adopt this coping strategy. Therefore, managers and supervisors should have an open communication about how to best use one's time in remote work. In an effort to bring together the many recommendations provided to managers and organizations during COVID-19 into a job demands and resources framework, Bilotta and co-authors (81) recommended that managers reduce emotional demands by creating a climate of authenticity, that could include training in the use of guilt-free breaks. Furthermore, employers should respect employees' right to disconnect (68). To achieve this, a transparent and clear communication of timelines and priorities from managers and supervisors is fundamental (81, 82). However, for university and public institution researchers, flexibility of working hours, autonomy and independence are cornerstones of their work activity. In this sector, limiting activity, including access to email, after formal working hours leads to reduced control over work, which can lead to negative consequences such as work-family conflict (83, 84). Therefore, training interventions to help individuals develop their skills in combining boundary management strategy with remote working may be more useful than fixed rules (85). Employers who value flexible working practices may benefit from adopting a person-centered approach that helps workers identify and monitor their own boundary management profile, providing personalized interventions while maintaining coordination across key organizational levels (68, 86).

In our sample, two types of conditions were significantly associated with the negative impact of WFH on interpersonal relationships: a) conditions related to the organization of the work space at home (living close to the workplace, the lack of a room in the house to dedicate to the office, often sharing the work room) and b) conditions related to the impossibility of practicing physical activity or cultivating hobbies as before the pandemic, or increased sedentary lifestyle. All these conditions point to the crisis of personal preferences for work/non-work boundary management induced by the obligation to work from home (85). As reported by Waismel-Manor et al. (83) “working from home involves changes in the material and symbolic nature of domestic space, producing a different spatial map of the household” and home workers can consider it necessary to have a separate work space at home. Indeed, this (re)organization of domestic environments leads other family members to consider that space as the worker's space and to respect it. In the absence of this reserved area, remote workers may face distractions from children or family members sharing their workspace (87), making it difficult to have a continuous workday and maintain a professional environment. Furthermore, having to show a private and informal environment during online meetings can be a source of fatigue and discomfort (loss of the right to privacy, sense of intrusion from supervisors and colleagues, loss of formality of the work environment, fear of intrusions from family members, need to position oneself in neutral areas of the house) and increase work-family conflict (88). Following the conservation of resources theory, Orellana et al. (76) showed that a higher resource loss derived from family-to-work conflict was associated with lower satisfaction not only in the receiving domain (work) but also in the origin domain of this loss or strain (family).

Differentiating even time as well as space can help remote workers better manage their work-life balance. Workers who find that going to the office is the best way to fulfill their segmentation preference may feel they never truly “leave” the office, as technology infiltration generally made work hours no longer confined to a 9-to-5 schedule. Despite the fact that the academic environment has long been accustomed to the use of technology after regular work hours (supplemental work), Mordi et al. (89) in their study based on spillover theory and work-life balance construct found that during the pandemic, academics in the UK experienced increased boundary permeability between work and non-work domains due to technology infiltration, and negative spillover as a consequence. That is, pre-pandemic remote working increased individuals' level of autonomy and flexibility, whereas pandemic conditions made boundary management difficult. Based on boundary theory, it has indeed been argued that perceived control of work may lead to more permeable boundaries in a remote setting and this may encourage supplemental work, ultimately increasing family-to-work conflict (84).

The sense of being “on call 24/7,” can impact family dynamics (54) as well as time for social commitments (90) and leisure (91). As stated before, stopping to engage in hobbies or playing physical activity during the WFH period was significantly associated with a negative impact of WFH on interpersonal relationships. A qualitative study based on work-life-balance construct and individual differences theory highlighted that hobbies can help workers to achieve moments of work-life balance and can be an influential antecedent of job satisfaction and commitment (92). Physical activity enhances self-efficacy, and individuals with strong self-efficacy beliefs about their ability to manage work and non-work responsibilities will, in turn, experience more satisfaction in both their work and family roles (93, 94).Consequently, organizations should foster supportive environments by actively promoting wellness programs that encourage physical activity (95).

WFH can benefit health and well-being by offering more opportunities to adopt healthier lifestyles—engaging in enjoyable activities, staying connected with friends and family, exercising regularly (even through short walks), eating healthily, and maintaining a regular sleep schedule. These habits positively impact productivity (19).

Viewing all our findings from a broad a job demand-resources perspective, as suggested by Demerouti and Bakker (10) in their new propositions in times of crises, this study reinforces that job characteristics alone do not fully explain employee well-being and motivation. Rather, the combined effect of demands and resources at individual, family, workplace, and organizational levels shapes these outcomes. Broadening the scope of interpretative models of work-life dynamics is also necessary because studies show that a one-size-fits-all approach is not feasible for flexible post-pandemic work arrangements (96), and that cultural and organizational adaptation are necessary (69).

This study has several limitations. First, while we assessed depression, we did not measure anxiety, which frequently co-occurs and significantly impacts mental health. Second, we did not directly assess work-life balance, which was only indirectly evaluated through questions about caregiving duties and the presence and characterization of cohabitants. Additional limitations have been described in our previous work (25), including the self-reported nature of the perceived impact of WFH, potential recall bias, self-selection of participants, low response rate, single research center, use of an ad-hoc questionnaire, potential exclusion of other relevant factors, and the lack of longer, more sophisticated tools to assess life domain issues. Finally, a couple of critical issues in the data analysis should be highlighted. The lack of statistical significance for some associations may result from the limited sample size, especially in the smaller subgroup. The width of some of the confidence intervals of the adjusted ORs in multiple regression models indicates inaccuracy of the estimates. Since the model diagnostics did not reveal algorithmic instability, this result is likely due to the fact that in each subgroup the outcomes that are not of interest in this study (a non-negative impact on interpersonal relationships, lack of change in depression severity, a non-negative impact on work experience) and their combinations are the most frequent. Therefore, the resulting contingency tables are skewed in favor of high ORs. The observed effect is likely real, but its precise magnitude is uncertain.

## 5 Conclusions

The evolving labor market poses challenges for everyone. To thrive in the post-coronavirus world, labor policies need enhancement. The analyses conducted in this study highlighted the relevance of issues related to health and well-being for a good work experience. This experiential knowledge can guide further research and the formulation of practices that foster healthy and productive remote working experiences. While our research was influenced by the emergency context in which our research was conducted, the implications of our analysis may contribute to creating a more sustainable model for “new normal” work practices. This could be particularly relevant in non-emergency periods when adequate time and preparations can prevent the repetition of critical behaviors.

## Data Availability

The datasets presented in this article are not readily available because the data used are pseudonymized and the CNR Research Ethics and Integrity Committee required that they are not made available. Requests to access the datasets should be directed to SS, saverio.sabina@cnr.it.

## In Memoriam

Giuseppe Ponzini, former colleague of the Institute for Research on Population and Social Policies, National Research Council, Brindisi, suddenly passed away on 9 July 2022.

## References

[1] Secretary-General of the OECD. Teleworking in the COVID-19 Pandemic: Trends and Prospects—OECD Policy Responses to Coronavirus (COVID-19) (2021). Available online at: https://www.oecd.org/en/publications/teleworking-in-the-covid-19-pandemic-trends-and-prospects_72a416b6-en.html (accessed November 6, 2024).

[2] Vyas L. “New normal” at work in a post-COVID world: work–life balance and labor markets. Policy Soc. (2022) 41:155–67. 10.1093/polsoc/puab011

[3] Ro C. Why the Future of Work Might Be ‘Hybrid' (2020). Available online at: https://www.bbc.com/worklife/article/20200824-why-the-future-of-work-might-be-hybrid (accessed November 6, 2024).

[4] Christian A. The Companies Sticking to Fully Remote Work. (2023). Available online at: https://www.bbc.com/worklife/article/20230912-the-companies-sticking-to-fully-remote-work (accessed November 6, 2024).

[5] Dawkins M. 20 Companies Embracing Permanent Remote Work-From-Home Jobs. (2024). Available online at: https://www.flexjobs.com/blog/post/companies-switching-remote-work-long-term/ (accessed November 6, 2024).

[6] Morse KF Fine PA Friedlander KJ. Creativity and leisure during COVID-19: examining the relationship between leisure activities, motivations, and psychological well-being. Front Psychol. (2021) 12:609967. 10.3389/fpsyg.2021.609967 34290635 PMC8288551

[7] Laß I Wooden M. Working from home and work–family conflict. Work Employ Soc. (2023) 37:176–95. 10.1177/09500170221082474

[8] Tessema MT Tesfom G Faircloth MA Tesfagiorgis M Teckle P. The “great resignation”: Causes, consequences, and creative HR management strategies. J Human Resour Sustain Stud. (2022) 10:161–78. 10.4236/jhrss.2022.101011

[9] Harford T. What Can We Learn From the Great WFH Experiment? (2020). Available online at: https://www.ft.com/content/0cb2ead1-59f8-4d59-9ed8-c76358f68bc4 (accessed November 6, 2024).

[10] Demerouti E Bakker AB. Job demands-resources theory in times of crises: New propositions. Organ Psychol Rev. (2023) 13:209–36. 10.1177/20413866221135022

[11] Innstrand ST Grødal K Christensen M. Balancing work and family life during the COVID-19 pandemic: exploring within and between group differences across time, gender, and worksites. Commun Work Family (2024) 14:1–20. 10.1080/13668803.2024.2399719

[12] Adams GA King LA King DW. Relationships of job and family involvement, family social support, and work–family conflict with job and life satisfaction. J Appl Psychol. (1996) 81:411. 10.1037/0021-9010.81.4.411

[13] Sim JYY Mustamil NM Wider W. Psychosocial working conditions and work engagement: the mediating role of psychological well-being. FWU J Soc Sci. (2023) 17:1–20. 10.51709/19951272/Winter2023/1

[14] Chambel MJ Castanheira F Santos A. Teleworking in times of COVID-19: the role of family-supportive supervisor behaviors in workers' work-family management, exhaustion, and work engagement. Int J Hum Resour Manage. (2023) 34:2924–59. 10.1080/09585192.2022.2063064

[15] Kundi YM Aboramadan M Elhamalawi EM Shahid S. Employee psychological well-being and job performance: exploring mediating and moderating mechanisms. Int J Organ Anal. (2020) 29:736–54. 10.1108/IJOA-05-2020-2204

[16] Tisu L Lupşa D Vîrgă D Rusu A. Personality characteristics, job performance and mental health: the mediating role of work engagement. Pers Individ Dif. (2020) 153:109644. 10.1016/j.paid.2019.10964428206838

[17] Urbina-Garcia A. What do we know about university academics' mental health? A systematic literature review. Stress Health. (2020) 36:563–85. 10.1002/smi.2956 32407607

[18] Bains N Abdijadid S. Major Depressive Disorder. Treasure Island (FL): StatPearls Publishing (2024). 32644504

[19] World Health Organization. Depressive Disorder (depression) (2023). Available online at: https://www.who.int/news-room/fact-sheets/detail/depression (accessed November 6, 2024).

[20] Evans-Lacko S Knapp M. Global patterns of workplace productivity for people with depression: absenteeism and presenteeism costs across eight diverse countries. Soc Psychiatry Psychiatr Epidemiol. (2016) 51:1525–37. 10.1007/s00127-016-1278-4 27667656 PMC5101346

[21] Lagerveld SE Bültmann U Franche RL van Dijk FJH Vlasveld MC van der Feltz-Cornelis CM. Factors associated with work participation and work functioning in depressed workers: a systematic review. J Occup Rehabil. (2010) 20:275–92. 10.1007/s10926-009-9224-x 20091105 PMC2923705

[22] Ekpanyaskul C Padungtod C. Occupational health problems and lifestyle changes among novice working-from-home workers amid the COVID-19 pandemic. Saf Health Work. (2021) 12:384–9. 10.1016/j.shaw.2021.01.010 33747597 PMC7954240

[23] Liu W Xu Y Ma D. Work-related mental health under COVID-19 restrictions: a mini literature review. Front Public Health. (2021) 9:788370. 10.3389/fpubh.2021.788370 34900925 PMC8651716

[24] Wang H Farokhnia F Sanchuli N. The effects of the COVID-19 pandemic on the mental health of workers and the associated social-economic aspects: A narrative review. Work. (2023) 74:31–45. 10.3233/WOR-220136 36245355

[25] Bodini A Leo CG Rissotto A Mincarone P Fusco S Garbarino S. The medium-term perceived impact of work from home on life and work domains of knowledge workers during COVID-19 pandemic: A survey at the National Research Council of Italy. Front Public Health. (2023) 11:1151009. 10.3389/fpubh.2023.1151009 36969653 PMC10036346

[26] Scoditti E Bodini A Sabina S Leo CG Mincarone P Rissotto A. Effects of working from home on lifestyle behaviors and mental health during the COVID-19 pandemic: a survey study. PLoS ONE. (2024) 19:e0300812. 10.1371/journal.pone.0300812 38558099 PMC10984516

[27] Garbarino S Bodini A Sabina S Leo CG Mincarone P Rissotto A. Not all workers experience equal sleep changes: insights from the “WorkInCovid” project. Clocks Sleep. (2025) 7:13. 10.3390/clockssleep7010013 40136850 PMC11941416

[28] García-Conesa M-T Philippou E Pafilas C Massaro M Quarta S Andrade V. Exploring the validity of the 14-Item Mediterranean diet adherence screener (MEDAS): a cross-national study in seven European countries around the Mediterranean Region. Nutrients (2020) 12:2960. 10.3390/nu12102960 32992649 PMC7601687

[29] Buysse DJ Reynolds 3rd CF Monk TH Berman SR Kupfer DJ. The Pittsburgh Sleep Quality Index: a new instrument for psychiatric practice and research. Psychiatry Res. (1989) 28:193–213. 10.1016/0165-1781(89)90047-4 2748771

[30] Curcio G Tempesta D Scarlata S Marzano C Moroni F Rossini PM. Validity of the Italian version of the Pittsburgh Sleep Quality Index (PSQI). Neurol Sci. (2013) 34:511–9. 10.1007/s10072-012-1085-y 22526760

[31] Johns MW A. new method for measuring daytime sleepiness: the Epworth sleepiness scale. Sleep. (1991) 14:540–5. 10.1093/sleep/14.6.540 1798888

[32] Vignatelli L Plazzi G Barbato A Ferini-Strambi L Manni R Pompei F. Italian version of the Epworth sleepiness scale: external validity. Neurol Sci. (2003) 23:295–300. 10.1007/s100720300004 12624716

[33] Sander C Hegerl U Wirkner K Walter N Kocalevent R-D Petrowski K. Normative values of the Epworth Sleepiness Scale (ESS), derived from a large German sample. Sleep Breath. (2016) 20:1337–45. 10.1007/s11325-016-1363-7 27234595

[34] Kocalevent R-D Hinz A Brähler E. Standardization of the depression screener patient health questionnaire (PHQ-9) in the general population. Gen Hosp Psychiatry. (2013) 35:551–5. 10.1016/j.genhosppsych.2013.04.006 23664569

[35] Bianchi R Verkuilen J Toker S Schonfeld IS Gerber M Brähler E. Is the PHQ-9 a unidimensional measure of depression? A 58,272-participant study. Psychol Assess. (2022) 34:595–603. 10.1037/pas0001124 35357877

[36] Kroenke K Spitzer RL Williams JB. The PHQ-9: validity of a brief depression severity measure. J Gen Intern Med. (2001) 16:606–13. 10.1046/j.1525-1497.2001.016009606.x 11556941 PMC1495268

[37] Mazzotti E Fassone G Picardi A Sagoni E Ramieri L Lega I. The patient health questionnaire (PHQ) for the screening of psychiatric disorders: a validation study versus the Structured Clinical Interview for DSM-IV axis I (SCID-I). J Psychopathol. (2003) 9:235–42.

[38] Core Team. R: A Language and Environment for Statistical Computing, Vienna (2022). Available online at: https://www.R-project.org

[39] Fox J Weisberg S. An {R} Companion to Applied Regression. Third Edition. Thousand Oaks: SAGE Publications, Inc. (2018). Available online at: https://uk.sagepub.com/en-gb/eur/an-r-companion-to-applied-regression/book246125

[40] Robin X Turck N Hainard A Tiberti N Lisacek F Sanchez J-C. pROC: an open-source package for R and S+ to analyze and compare ROC curves. BMC Bioinform. (2011) 12:77. 10.1186/1471-2105-12-77 21414208 PMC3068975

[41] Lele SR Keim JL Sólymos P. Resource Selection (Probability) Functions for Use-Availability Data (2023). Available online at: https://cran.r-project.org/web/packages/ResourceSelection/index.html

[42] de Oliveira C Saka M Bone L Jacobs R. The role of mental health on workplace productivity: a critical review of the literature. Appl Health Econ Health Policy. (2023) 21:167–93. 10.1007/s40258-022-00761-w 36376610 PMC9663290

[43] Joyce S Modini M Christensen H Mykletun A Bryant R Mitchell PB. Workplace interventions for common mental disorders: a systematic meta-review. Psychol Med. (2016) 46:683–97. 10.1017/S0033291715002408 26620157

[44] Carolan S Harris PR Greenwood K Cavanagh K. Increasing engagement with an occupational digital stress management program through the use of an online facilitated discussion group: Results of a pilot randomised controlled trial. Internet Interv. (2017) 10:1–11. 10.1016/j.invent.2017.08.001 30135747 PMC6084816

[45] Goetzel RZ Roemer EC Holingue C Fallin MD McCleary K Eaton W. Mental health in the workplace: a call to action proceedings from the mental health in the workplace-public health summit. J Occup Environ Med. (2018) 60:322–30. 10.1097/JOM.0000000000001271 29280775 PMC5891372

[46] Wu A Roemer EC Kent KB Ballard DW Goetzel RZ. Organizational best practices supporting mental health in the workplace. J Occup Environ Med. (2021) 63:e925–31. 10.1097/JOM.0000000000002407 34840320 PMC8631150

[47] Muñoz RF Cooper LA. The COVID-19 pandemic and mental health-implementing evidence-based interventions to advance equity and reverse a worsening crisis. JAMA Health Forum. (2022) 3:e221282. 10.1001/jamahealthforum.2022.1282 36218968

[48] Furukawa TA Horikoshi M Kawakami N Kadota M Sasaki M Sekiya Y. Telephone cognitive-behavioral therapy for subthreshold depression and presenteeism in workplace: a randomized controlled trial. PLoS ONE. (2012) 7:e35330. 10.1371/journal.pone.0035330 22532849 PMC3330821

[49] Sanderson K Tilse E Nicholson J Oldenburg B Graves N. Which presenteeism measures are more sensitive to depression and anxiety? J Affect Disord. (2007) 101:65–74. 10.1016/j.jad.2006.10.024 17156851

[50] Johnston DA Harvey SB Glozier N Calvo RA Christensen H Deady M. The relationship between depression symptoms, absenteeism and presenteeism. J Affect Disord. (2019) 256:536–40. 10.1016/j.jad.2019.06.041 31280078

[51] Kang M Lee W-T Yun B Yoon J-H. Association between sickness presenteeism and depressive symptoms by occupation and employment type during the COVID-19 pandemic. Safety Health Work. (2024) 15:338–44. 10.1016/j.shaw.2024.06.002 39309283 PMC11410493

[52] Magalhães S Barbosa J Borges E. The relationship between presenteeism, quality of life and social support in higher education professionals: a cross-sectional path analysis. PLoS ONE. (2022) 17:e0267514. 10.1371/journal.pone.0267514 35446913 PMC9022867

[53] Schmitz H Bauer JF Niehaus M. Working anytime and anywhere -even when feeling ill? A cross-sectional study on presenteeism in remote work. Safety Health Work. (2023) 14:375–83. 10.1016/j.shaw.2023.11.001 38187205 PMC10770276

[54] Kim J Henly JR Golden LM Lambert SJ. Workplace flexibility and worker well-being by gender. J Marr Family. (2020) 82:892–910. 10.1111/jomf.12633

[55] Karanika-Murray M Biron C. The health-performance framework of presenteeism: towards understanding an adaptive behaviour. Human Relations. (2020) 73:242–61. 10.1177/0018726719827081

[56] Bonaccio S Lapierre LM O'Reilly J. Creating work climates that facilitate and maximize the benefits of disclosing mental health problems in the workplace. Organ Dyn. (2019). 10.1016/j.orgdyn.2019.03.006

[57] Parikh H. How Remote Work Can Impact Employees' Mental Health (2023). Available online at: https://www.forbes.com/sites/forbeshumanresourcescouncil/2023/07/03/how-remote-work-can-impact-employees-mental-health/ (accessed March 11, 2025).

[58] Nelson C. Supporting Mental Health in the Workforce (2023). Available online at: https://www.ibiweb.org/resources/supporting-mental-health-in-the-workplace?hsLang=en# (accessed March 11, 2025).

[59] Brenner B. Understanding Work Depression (2023). Available online at: https://therapygroupdc.com/therapist-dc-blog/understanding-work-depression/ (accessed March 11, 2025).

[60] Ferreira AI Mach M Martinez LF Miraglia M. Sickness presenteeism in the aftermath of COVID-19: is presenteeism remote-work behavior the new (ab)normal? Front Psychol. (2022) 12:748053. 10.3389/fpsyg.2021.748053 35153891 PMC8830031

[61] Winwood PC Bakker AB Winefield AH. An investigation of the role of non-work-time behavior in buffering the effects of work strain. J Occup Environ Med. (2007) 49:862–71. 10.1097/JOM.0b013e318124a8dc 17693784

[62] Abdel Hadi S Bakker AB Häusser JA. The role of leisure crafting for emotional exhaustion in telework during the COVID-19 pandemic. Anxiety Stress Coping. (2021) 34:530–44. 10.1080/10615806.2021.1903447 33769142

[63] Wang J Xiong Y Murad M Chaudhary NI Waqar H. Role of online time-spatial job crafting and leisure crafting on remote work performance through tele-pressure and techno-self-efficacy. Sustainability. (2023) 15:11936. 10.3390/su151511936

[64] Fancourt D Finn S. What is the evidence on the role of the arts in improving health and well-being? A scoping review. Health Evidence Network synthesis report 67 (2019). Copenhagen: WHO Regional Office for Europe. Available online at: https://europepmc.org/article/NBK/nbk553773 (accessed March 10, 2025). 32091683

[65] Pressman SD Matthews KA Cohen S Martire LM Scheier M Baum A. Association of enjoyable leisure activities with psychological and physical well-being. Biopsychosoc Sci Med. (2009) 71:725-32. 10.1097/PSY.0b013e3181ad7978 19592515 PMC2863117

[66] Stone L Ozimek A. Early Remote Work Impacts on Family Formation (2023). Available online at: https://eig.org/remote-work-family-formation/ (accessed November 6, 2024).

[67] Barhate B Dirani KM. Career aspirations of generation Z: a systematic literature review. Eur J Train Dev. (2022) 46:139–57. 10.1108/EJTD-07-2020-0124

[68] Henke JB Jones SK O'Neill TA. Skills and abilities to thrive in remote work: what have we learned. Front Psychol. (2022) 13:893895. 10.3389/fpsyg.2022.893895 36600705 PMC9807077

[69] Torres S Orhan MA. How it started, how it's going: Why past research does not encompass pandemic-induced remote work realities and what leaders can do for more inclusive remote work practices. Psychol Leader Leadersh. (2023) 26:1. 10.1037/mgr0000135

[70] Luong A Rogelberg SG. Meetings and more meetings: The relationship between meeting load and the daily well-being of employees. Group Dyn Theor Res Pract. (2005) 9:58. 10.1037/1089-2699.9.1.58

[71] Beyea D Lim C Lover A Foxman M Ratan R Leith A. Zoom fatigue in review: a meta-analytical examination of videoconferencing fatigue's antecedents. Comput Human Behav Rep. (2025) 17:100571. 10.1016/j.chbr.2024.100571

[72] Patil S Janani N Anand Kumar P Shobitha J Nagaprakash T. The impact of remote work on work-life balance and employee productivity. J Res Admin. (2024) 6:658–69.

[73] Delanoeije J Verbruggen M. Between-person and within-person effects of telework: a quasi-field experiment. Eur J Work Organ Psychol. (2020) 29:795–808. 10.1080/1359432X.2020.1774557

[74] Yucel D Chung H. Working from home, work–family conflict, and the role of gender and gender role attitudes. Commun Work Fam. (2023) 26:190–221. 10.1080/13668803.2021.1993138

[75] Voydanoff P. Work demands and work-to-family and family-to-work conflict: direct and indirect relationships. J Fam Issues. (2005) 26:707–26. 10.1177/0192513X05277516

[76] Orellana L Schnettler B Miranda-Zapata E Saracostti M Poblete H Lobos G. Job satisfaction as a mediator between family-to-work conflict and satisfaction with family life: a dyadic analysis in dual-earner parents. Appl Res Qual Life. (2023) 18:491–520. 10.1007/s11482-022-10082-8 35966805 PMC9361244

[77] Schnettler B Miranda-Zapata E Orellana L Saracostti M Poblete H Lobos G. Intra- and inter-individual associations of family-to-work conflict, psychological distress, and job satisfaction: gender differences in dual-earner parents during the COVID-19 pandemic. Behav Sci. (2024) 14:56. 10.3390/bs14010056 38247708 PMC10813670

[78] Schnettler B Miranda-Zapata E Orellana L Saracostti M Poblete H Concha-Salgado A. Family-to-work conflict linked to psychological distress and family life satisfaction during the second year of the COVID-19 pandemic in dual-earner parents with adolescents. Front Public Health. (2024) 12:1476549. 10.3389/fpubh.2024.1476549 39678244 PMC11638038

[79] Wu H Song QC Proctor RW Chen Y. Family relationships under work from home: exploring the role of adaptive processes. Front Public Health. (2022) 10:782217. 10.3389/fpubh.2022.782217 35372190 PMC8965466

[80] Perry SJ Carlson DS Kacmar KM Wan M. (Maggie), Thompson MJ. Interruptions in remote work: a resource-based model of work and family stress. J Bus Psychol. (2023) 38:1023–41. 10.1007/s10869-022-09842-y 36189432 PMC9510213

[81] Bilotta I Cheng S Davenport MK King E. Using the job demands-resources model to understand and address employee well-being during the COVID-19 pandemic. Ind Organ Psychol. (2021) 14:267–73. 10.1017/iop.2021.43

[82] Chuang Y-T Chiang H-L Lin A-P. Information quality, work-family conflict, loneliness, and well-being in remote work settings. Comput Human Behav. (2024) 154:108149. 10.1016/j.chb.2024.108149

[83] Waismel-Manor R Wasserman V Shamir-Balderman O. No room of her own: married couples' negotiation of workspace at home during COVID-19. Sex Roles. (2021) 85:636–49. 10.1007/s11199-021-01246-1 34629688 PMC8493050

[84] Kost D Kopperud K Buch R Kuvaas B Olsson UH. The competing influence of psychological job control on family-to-work conflict. J Occup Organ Psychol. (2023) 96:351–77. 10.1111/joop.1242635139124

[85] Allen TD Merlo K Lawrence RC Slutsky J Gray CE. Boundary management and work-nonwork balance while working from home. Appl Psychol. (2021) 70:60–84. 10.1111/apps.12300

[86] Kossek EE Ruderman MN Braddy PW Hannum KM. Work–nonwork boundary management profiles: A person-centered approach. J Vocat Behav. (2012) 81:112–28. 10.1016/j.jvb.2012.04.00337450353

[87] Zhang S Moeckel R Moreno AT Shuai B Gao J A. work-life conflict perspective on telework. Transp Res Part A Policy Pract. (2020) 141:51–68. 10.1016/j.tra.2020.09.007 32982087 PMC7509537

[88] Li BJ Malviya S Tandoc Jr EC. Videoconferencing and work-family conflict: exploring the role of videoconference fatigue. Commun Stud. (2022) 73:544–60. 10.1080/10510974.2022.2153894

[89] Mordi C Ajonbadi HA Adekoya OD. Technology infiltration: permeable boundaries and work–life spillover experiences among academics in the United Kingdom during the COVID-19 pandemic. Pers Rev. (2024) 53:1269–88. 10.1108/PR-10-2022-0693

[90] Lunau T Bambra C Eikemo TA van der Wel KA Dragano N A. balancing act? Work–life balance, health and well-being in European welfare states. Eur J Public Health. (2014) 24:422–7. 10.1093/eurpub/cku010 24567294

[91] Allen TD Herst DE Bruck CS Sutton M. Consequences associated with work-to-family conflict: a review and agenda for future research. J Occup Health Psychol. (2000) 5:278–308. 10.1037/1076-8998.5.2.278 10784291

[92] Campbell-Nowlin M. Exploring Hobbies as a Critical Component of Work-Life Balance: Perceptions of Their Influence on Job Satisfaction and Job Performance (doctoral dissertations and projects) (2024), 5913. Available online at: https://digitalcommons.liberty.edu/doctoral/5913 (accessed March 10, 2025).

[93] Chan XW Kalliath T Brough P Siu O-L O'Driscoll MP Timms C. Work–family enrichment and satisfaction: the mediating role of self-efficacy and work–life balance. Int J Human Resour Manage. (2016) 27:1755–76. 10.1080/09585192.2015.1075574

[94] Chan XW Kalliath T Brough P O'Driscoll M Siu O-L Timms C. Self-efficacy and work engagement: test of a chain model. Int J Manpow. (2017) 38:819–34. 10.1108/IJM-11-2015-0189

[95] Deepa R Dharshini JJ. Driving sustained work engagement: moderating role of leadership and organizational support for remote work. Manage Res Rev. (2024) 47:464–82. 10.1108/MRR-11-2022-0806

[96] Bonner C. Making Post-Pandemic Hybrid & Flexible Arrangements Work (2023). Available online at: https://www.ibiweb.org/resources/making-post-pandemic-hybrid-flexible-arrangements-work?hsLang=en# (accessed November 6, 2024).

[97] Bonda J Morrow C B Gueorguieva R Brown M Hawrilenko M Krystal JH. Clinical and financial outcomes associated with a workplace mental health program before and during the COVID-19 pandemic. JAMA Netw. Open (2022) 5:e2216349. 10.1001/jamanetworkopen.2022.16349 35679044 PMC9185188

