# Supplementary material for: Mental health and social relationships shape the work-from-home experience: lessons from COVID-19 pandemic
Source: Front Public Health. 2025 Apr 10;13:1526885. doi: 10.3389/fpubh.2025.1526885 (PMC12018375; doi:10.3389/fpubh.2025.1526885)
Supplement: Supplementary file 1 [file Data_Sheet_1.docx]

Supplementary Material

# Participant’s general characteristics

Supplementary Table 1 – General demographic and working characteristics of the sample and comparison with the source population as of December 2021, 31st. Source CNR.

|  | Source  Population  $n=8543$ | Sample  $n=748$ | |
| --- | --- | --- | --- |
|  | % | N | % |
| ***Gender*** | | | |
| Man | 53.0 | 317 | 42.4 |
| Woman | 47.0 | 425 | 57.6 |
| ***Age group* (*years*)** | | | |
| ≤39 | 14.9 | 90 | 12.0 |
| 40-49 | 35.1 | 275 | 36.8 |
| 50-59 | 35.3 | 285 | 38.1 |
| ≥60 | 14.7 | 98 | 11.9 |
| ***Living status*** | | | |
| Living alone | NA | 108 | 14.4 |
| Married or living together,  no children | NA | 358 | 47.9 |
| Married or living together,  with children | NA | 282 | 37.7 |
| ***Italian macro-region of residence*** | | | |
| North | 24.0 | 244 | 32.6 |
| Center | 40.4 | 261 | 34.9 |
| South | 25.1 | 168 | 22.5 |
| Islands | 10.5 | 75 | 10.0 |
| ***Education level*** | | | |
| Graduation | NA | 614 | 82.1 |
| No graduation | NA | 134 | 17.9 |
| ***Professional profile**** | | | |
| Amministrative staff | 9.6 | 75 | 9.2 |
| Technical staff | 27.4 | 163 | 7.0 |
| Technologist | 9.1 | 77 | 9.9 |
| Researcher | 51.1 | 433 | 9.9 |
| * 3.3% of officials were not involved in the questionnaire  as they have a different work regime. | | | |

# Variable of interest

Supplementary Table 2 – Normative thresholds, adopted thresholds and relevant variations in the total scores considered in the analysis.

| **Indicator** | **Normative Thresholds**  **of total scores** | **Adopted Thresholds/significant variations** |
| --- | --- | --- |
| **Patient Health Questionnaire**  **PHQ-9** | **Minimal level of d.s.,**  **0-4** | **Minimal, 0-4** |
|  | **Mild, 5-9** | **Mild+, 5-27** |
|  | **Moderate, 10-14** |  |
|  | **Moderately severe, 15-19** |  |
|  | **Severe, 20-27** |  |
| Pittsburgh Sleep Quality Index - **PSQI** | **Good sleep, 0-5** | **Good sleep, 0-5** |
|  | **Poor sleep, 6-21** | **Poor sleep, 6-21** |
| Mediterranean Diet Adherence Score  **MEDAS** | **weak adherence, 0-5** | **Increased score during WFH (Improved adherence)**  **Decreased score during WFH (worsened adherence)** |
|  | **moderate to fair adherence, 6–9** |  |
|  | **good or very good adherence 10-14** |  |

**Legend. d.s.: depression severity.** WFH: working from home*.*

Supplementary Table 3 – Questions, available answers and aggregations considered in the analysis.

| **Question** | **Original scale** | **Scale used for the analysis** |
| --- | --- | --- |
| **Has the transition to WFH changed your body weight?** | No, my weight is stable; | Unchanged |
|  | Yes, I have lost weight; | Decreased |
|  | Yes, I have gained some weight; | Increased |
|  | Yes, I have gained a lot of weight |  |
| **Has the time you usually spend sitting or lying down during the day (including time spent working) changed compared to before you started WFH?** | **Much decreased** | **Decreased** |
|  | **Decreased** |  |
|  | **Unchanged** | **Unchanged** |
|  | **Increases** | **Increased** |
|  | **Much increased** |  |
| **In your opinion, how has the experience of WFH during the pandemic affected the quality of interpersonal relationships within your family?(*)** | **Very negatively** | **Negative** |
|  | **Negatively** |  |
|  | **No effect** | **Null or positive** |
|  | **Positively** |  |
|  | **Very positively** |  |
| **In your opinion, how has the experience of WFH during the pandemic affected the quality of interpersonal relationships within your network of friends?(*)** | **Very negatively** | **Negative** |
|  | **Negatively** |  |
|  | **No effect** | **Null or positive** |
|  | **Positively** |  |
|  | **Very positively** |  |
| **Has the transition to WFH changed your hobbies/pastimes (excluding physical activity)?** | **I have never had hobbies/pastimes** | **Habit of hobbies/pastimes during WFH: No** |
|  | **I had hobbies/pastimes but not now** |  |
|  | **I did not have but have now** | **Habit of hobbies/pastimes during WFH: Yes** |
|  | **I had and still have hobbies/pastimes** |  |
| **How many days did you return to the office during the entire pandemic period?** | **<20** | **Number of days of work in presence:** |
|  | **21-60** | **≤ 60** |
|  | **61-120** | **> 60** |
|  | **>120** |  |
| **How long did it take you on average to get to work before the current work-from-home situation?** | **≤ 15 minutes** | **Commuting time:** |
|  | **15-30 minutes** | **≤ 15 minutes** |
|  | **30-60 minutes** | **15-30 minutes** |
|  | **>60 minutes** | **>30 minutes** |

**Legend.** WFH: working from home*.* (***) The answers to these two questions were combined to create the variable "Changes in interpersonal relationships within family or friends due to WFH." The result was defined by considering only the worst of the two answers, on the original scale.**

# Bivariate analysis in subgroups

Supplementary Table 4 – Chi-square test for the association of the binary impact of WFH on the work experience with meaningful changes of health-related variables in the two subgroups of Minimal and Mild+ depression severity. The sub-groups analysis refers to the 733 subjects with complete data.

| **Changes in:** | **Meaningful changes of health-related variables** | **Impact of WFH on work experience** | | | ***p-value*** |
| --- | --- | --- | --- | --- | --- |
|  |  | **Negative**  **(WEM<3)** | **Positive**  **(WEM ≥ 3)** | |  |
| ***Minimal* subgroup (N = 489)** | | | | | |
| **Depression severity** | PHQ-9 from ≤ 4 to > 4  (*Worsening*) | 56.7% | 43.2% | | $\ll$0.001 |
|  | Unchanged, PHQ-9 ≤ 4 | 21.7% | 78.3% | |  |
| **Presence of**  **difficulties brought by d.s.*** | Unchanged, *No* | 20.7% | 79.4% | | $\ll$0.001 |
|  | Unchanged, *Yes* | 50.0% | 50.0% | |  |
|  | From *No* to *Yes* | 59.1% | 40.9% | |  |
|  | From *Yes* to *No* | 3.4% | 96.6% | |  |
| **Adherence to MD** | Not increased | 33.7% | 66.3% | | 0.03 |
|  | Increased | 24.0% | 76.0% | |  |
| **Sleep quality** | Unchanged, PSQI > 5  (*poor*) | 28.6% | 71.4% | | <0.001 |
|  | Unchanged, PSQI ≤ 5  (*good*) | 28.3% | 71.7% | |  |
|  | PSQI from ≤ 5 to > 5  (*Worsening*) | 48.5% | 51.5% | |  |
|  | PSQI from > 5 to ≤ 5  (*Improving*) | 13.0% | 87.0% | |  |
| **Weight** | Unchanged | 26.0% | 74.0% | | 0.02 |
|  | Increased | 37.0% | 63.0% | |  |
|  | Decreased | 22.2% | 77.8% | |  |
| **Sedentary lifestyle** | Unchanged | 19.1% | | 80.9% | $\ll$0.001 |
|  | Increased | 41.4% | | 58.6% |  |
|  | Decreased | 20.8% | | 79.2% |  |
| **Interpersonal relationships within family or friends due to WFH** | Negative impact | 73.7% | 26.3% | | $\ll$0.001 |
|  | Null or positive | 19.8% | 80.2% | |  |
| **Hobby and pastimes** | I have never had hobbies/pastimes | 28.8% | 71.2% | | 0.008 |
|  | I did not have but have now | 15.4% | 84.6% | |  |
|  | I had hobbies/pastimes but not now | 50.0% | 50.0% | |  |
|  | I had and still have hobbies/pastimes | 29.1% | 70.9% | |  |
| ***Mild+* subgroup (N = 244)** | | | | | |
| **Depression severity** | Unchanged, PHQ-9 > 4 | 35.3% | 64.7% | | <0.001 |
|  | PHQ-9 from > 4 to ≤ 4  (*Improving*) | 10.4% | 89.6% | |  |
| **Presence of**  **difficulties brought by d.s.**** | Unchanged, *No* | 20.9% | 79.1% | | <0.001 |
|  | Unchanged, *Yes* | 35.7% | 64.3% | |  |
|  | From *No* to *Yes* | 71.4% | 28.6% | |  |
|  | From *Yes* to *No* | 7.3% | 92.7% | |  |
| **Adherence to MD** | Not increased | 29.6% | 70.4% | | 0.48 |
|  | Increased | 24.8% | 75.2% | |  |
| **Sleep quality** | Unchanged, PSQI > 5  (*poor*) | 29.7% | 70.3% | | 0.053 |
|  | Unchanged, PSQI ≤ 5  (*good*) | 20.9% | 79.1% | |  |
|  | PSQI from ≤ 5 to > 5  (*Worsening*) | 52.9% | 47.1% | |  |
|  | PSQI from > 5 to ≤ 5  (*Improving*) | 26.0% | 74.0% | |  |
| **Weight** | Unchanged | 20.2% | 79.8% | | 0.11 |
|  | Increased | 33.6% | 66.4% | |  |
|  | Decreased | 26.7% | 73.3% | |  |
| **Sedentary lifestyle** | Unchanged | 16.7% | 83.3% | | 0.002 |
|  | Increased | 37.3% | 62.7% | |  |
|  | Decreased | 17.5% | 82.5% | |  |
| **Interpersonal relationships within family or friends due to WFH** | Negative | 59.2% | 40.8% | | $\ll$0.001 |
|  | Null or positive | 19.5% | 80.5% | |  |
| **Hobby and pastimes** | I have never had hobbies/pastimes | 43.6% | 56.4% | | 0.001 |
|  | I did not have but have now | 12.5% | 87.5% | |  |
|  | I had hobbies/pastimes but not now | 52.6% | 47.4% | |  |
|  | I had and still have hobbies/pastimes | 22.8% | 77.2% | |  |

Legend. *Minimal* subgroup: participants with total score pre-pandemic of PHQ-9 ≤ 4; *Mild*+ subgroup: participants with total pre-pandemic score of PHQ-9 > 4; d.s.: depressive symptoms; MD: Mediterranean diet. WEM: work experience measure. (*) The considered participants were 375, of whom 124 had WEM <3 and 251 had WEM ≥3. (**) The considered participants were 231, of whom 67 had WEM <3 and 164 had WEM ≥3.

# Moderation Analysis


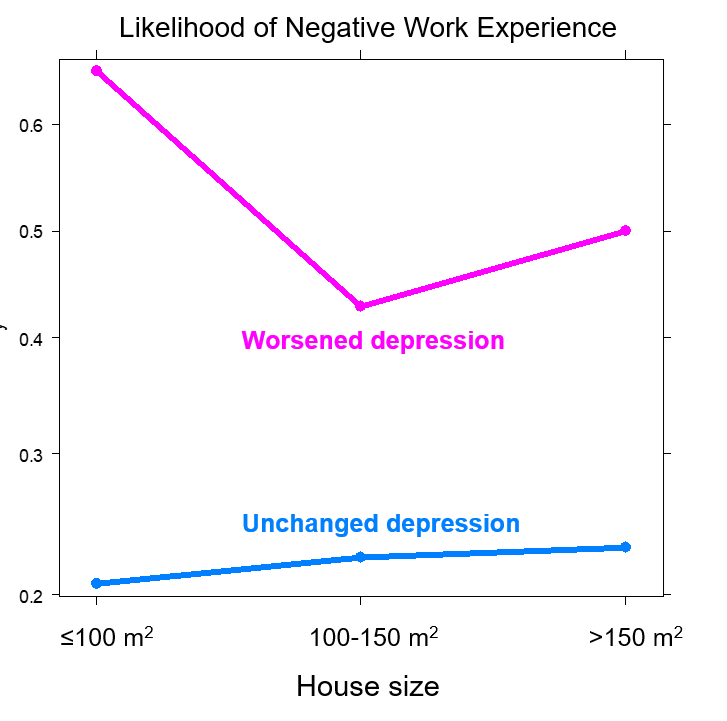


Supplementary Figure 1 - MINIMAL subgroup. Among those who reported worsening depression severity, those who lived in smaller homes (≤100 m^2^) were more likely to rate the impact of working from home on their work experience negatively.
